# Comparison of Proximal Caries Detection in Primary Teeth between Laser Fluorescence and Bitewing Radiography: An *in vivo* Study

**DOI:** 10.5005/jp-journals-10005-1257

**Published:** 2015-02-09

**Authors:** Ratheesh Mepparambath, Sham S Bhat, Sundeep K Hegde, G Anjana, M Sunil, Sherryl Mathew

**Affiliations:** Senior Lecturer, Department of Pedodontics and Preventive Dentistry, Royal Dental College, Palakkad, Kerala, India; Professor and Head, Department of Pedodontics and Preventive Dentistry Yenepoya Dental College, Mangalore, Karnataka, India; Professor, Department of Pedodontics and Preventive Dentistry Yenepoya Dental College, Mangalore, Karnataka, India; Professor and Head, Department of Pedodontics and Preventive Dentistry, Royal Dental College, Palakkad, Kerala, India; Professor, Department of Pedodontics and Preventive Dentistry, Royal Dental College, Palakkad, Kerala, India; Senior Lecturer, Department of Pedodontics and Preventive Dentistry, Royal Dental College, Palakkad, Kerala, India

**Keywords:** DIAGNOdent, Bitewing radiograph, Proximal caries detection, Primary teeth, Dentin caries, Enamel caries.

## Abstract

**Background and objective:** Proximal caries detection is of great importance because of the rapid rate of caries progression and the difficulty in determining the absence or presence of a lesion in primary dentition. Although, various methods for caries diagnosis offer good diagnostic performances, they provide varying sensitivities for detecting proximal carious lesions. The objective of this study was to compare, *in vivo*, the accuracy of DIAGNOdent and bitewing radiography at detecting proximal caries in primary teeth.

**Materials and methods:** One Hundred and one primary maxillary and mandibular molars without obvious cavities of children between the age group of 3 and 10 years were included. The teeth were first subjected to DIAGNOdent examination followed by bitewing radiography. The specificity and sensitivity of the systems were calculated.

**Results:** At the dentin caries (D3) level, the sensitivity of DIAGNOdent and bitewing radiography was 78.5%; at the enamel caries (D1 and D2) level it was 39.12% and for the sound teeth (D0) it was found to be 76.52%. A strong association was observed between the DIAGNOdent and the bitewing radiograph (p < 0.001).

**Conclusion:** The DIAGNOdent can be used as an alternative diagnostic method in detection of proximal caries in primary teeth. But when seen at the each caries level, the DIAGNOdent is more accurate at the D0 and D3 threshold.

**How to cite this article:** Mepparambath R, Bhat SS, Hegde SK, Anjana G, Sunil M, Mathew S. Comparison of Proximal Caries Detection in Primary Teeth between Laser Fluorescence and Bitewing Radiography: An *in vivo* Study. Int J Clin Pediatr Dent 2014;7(3):163-167.

## INTRODUCTION

Among the different types of carious lesions, proximal caries is notorious for its rapid rate of progression and the difficulty in determining the presence of the lesion.^1-3^ It has been established by various authors that the radio-graphic techniques are superior over clinical examination in the detection of interproximal carious lesions.^4-8^

But, bitewing radiographs, which is the most common method used to diagnose proximal caries, exposes the patient to a relatively high dose of ionizing radiation.^9^ Alternative methods to detect proximal caries, like the DIAGNOdent, are expected to overcome the disadvantages of these conventional methods.^10^

Hibst and Paulus^11^ discovered that bacterial metabolites within caries produce fuorescence that can be enhanced by a laser light. Based on this, the DIAGNOdent (KaVo, Biberach, Germany), a portable laser diode based device was developed.^12^ DIAGNOdent's accuracy has been studied both *in vitro* and *in vivo* for occlusal caries in primary and permanent teeth .^10,13-15^ Several investigations indicate that DIAGNOdent could be applied for use on smooth surface caries and proximal caries of permanent teeth.^10,16-18^ The results of these studies show that DIAGNOdent is accurate and reproducible in caries detection. But, only a few studies have indicated its use on the proximal surfaces of primary teeth.^10^

Therefore, the aim of our study was to compare, *in vivo*, accuracy of DIAGNOdent and bitewing radiography at detecting proximal caries in primary teeth.

## MATERIALS AND METHODS

The study was reviewed and approved by the Ethics Committee of Yenepoya University, Mangalore, India.

### Patient Selection

One hundred and one deciduous maxillary and mandi-bular molars without obvious cavities were identified in children between the age group of 3 to 10 years who had reported to the Department of Pedodontics and Preventive Dentistry, Yenepoya Dental College and Hospital, Mangalore, India. Once the prospective patients were identified, the procedure and aims of the study were explained to them or their guardian and informed consent was obtained.

The following inclusion criteria were used to determine entry into the study: the included teeth must be deciduous maxillary or mandibular molars; the tooth must be intact or cavities if present should be less than 1 mm in diameter on the proximal surface. Teeth with frank cavitation or symptoms of pulpitis; presence of proximal restorations or hypoplastic pits were excluded from participation in the study.

### DIAGNOdent Examination

As per the manufacturer's instructions, calibration of the DIAGNOdent (KaVo, Biberach, Germany) was performed with a ceramic standard provided by the company for every patient. Following the tooth selection, oral prophylaxis was performed and polishing of the teeth were done using rubber cup and pumice/water slurry. The preselected teeth were air dried using a three way syringe and the Probe A of the DIAGNOdent was used for the examination. The tip of the laser device was then placed perpendicular to the surface being examined, i.e. at the marginal ridge of the occlusal surface and at the buccal and lingual embrasures.

The DIAGNOdent device has two displays: one shows the moment reading and the other shows the peak reading. The moment reading indicates the real time value that the probe tip measures during the scanning of the surface. The peak reading refers to the highest level scanned on the tooth surface.

The peak reading for each tooth was recorded. The readings were then interpreted according to the criteria put forward by the manufacturer (KaVo, 2001) (Table 1).

**Table 1:** **Table 1:** Caries interpretation based on the criteria put forward by the manufacturer (KaVo, 2001)

*Caries level*		*Interpretation*	
Sound (D0)		0-9	
Enamel caries (D1-D2)		10-17	
Dentin caries (D3)		18-99	

**Table 2:** **Table 2:** Radiographic interpretation based on criteria put forward by Pitts (1984)^33^

*Score*		*Caries level*		*Criteria*	
R0		D0-Sound		No Radiolucency	
R1		D1-Initial caries		Zone of increased radiolucency, confined to outer half of enamel	
R2		D2-Enamel caries		Zone of increased radiolucency involving both inner and outer halves of enamel layer, including lesions extending up to but not beyond the DEJ	
R3		D3-Dentin caries		Zone of increased radiolucency penetrating the enamel and DEJ and progressing into the dentin	

**Table 3:** **Table 3:** Total number of teeth examined

*Teeth examined*		*First molar*		*Second molar*		*Total*	
Maxillary teeth		25		24		49	
Mandibular teeth		26		26		52	
Total		51		50		101	

**Table 4:** **Table 4:** Total number of teeth surfaces examined

*Teeth examined*		*First molar*		*Second molar*		*Total*	
Maxillary teeth		38		42		79	
Mandibular teeth		43		46		90	
Total		81		88		169	

**Table 5:** **Table 5:** Caries distribution as per DIAGNOdent interpretation

*Criteria*		*Caries level*		*No. of teeth*	
Sound (0-9)		D0		110	
Enamel caries (10-17)		D1 + D2		34	
Dentin caries (18-99)		D3		25	
Total				169	

### Bitewing Radiography

Once the DIAGNOdent recordings of the teeth were completed, the bitewing radiographs were taken under standardized conditions. A bitewing holder was used to hold the radiograph in position. If any defect such as overlapping of the teeth occurred, the radiographs were repeated.

The radiographs taken were interpreted as per the criteria put forward by Pitts (1984)^19^ (Table 2).

The obtained results were statistically analyzed to obtain the sensitivity and specificity using the Chi-square test.

## RESULTS

A total of 101 maxillary and mandibular primary molars without obvious cavities were identified in children between the age group of 3 and 10 years. The distribution of the teeth examined is illustrated as shown in Table 3.

Of the 101 teeth selected, the total surfaces examined were 169. Tooth surface without contact to the adjacent tooth was not examined. Table 4 illustrates the distribution of the teeth surfaces examined.

Of the 169 teeth surfaces examined, the DIAGNOdent interpretation showed 110 teeth surfaces to have no caries (sound), 34 surfaces as enamel caries and 25 surfaces as dentinal caries. Table 5 illustrates the DIAGNOdent interpretation.

Of the 169 teeth surfaces interpreted by radiographs, 132 teeth surfaces showed no radiolucency, 17 surfaces showed zone of radiolucency confined to the outer half of the enamel, 6 teeth surfaces showed zone of radiolucency involving both the inner and outer halves of the enamel and 14 surfaces showed radiolucency extending beyond the Dentinoenamel junction. Table 6 illustrates the radiographic interpretation based on the level of caries.

The statistical analysis correlating the interpretations obtained from DIAGNOdent and bitewing radiograph has been elaborated in Table 7.

Of the 110 teeth surfaces (65.1%) scored as sound (D0) on the DIAGNOdent as against 101 surfaces (76.5%) were radiographically sound. Seven teeth surfaces (41.2%) recorded as sound by DIAGNOdent showed caries confning to the outer half of the enamel and one tooth surface (16.7%) showed caries involving both the inner and outer halves of the enamel, indicating enamel caries radiographically. One tooth surface (7.1%) recorded as sound by the DIAGNOdent showed radiolucency extending beyond the dentinoenamel junction, indicating dentinal caries. The sensitivity of the DIAGNOdent technique at the D0 threshold was found to be 76.52% and the specificity was 75.68%.

Of the 34 teeth surfaces (20.1%) scored as enamel caries (D1, D2) by the DIAGNOdent, nine teeth surfaces were found to have radiolucencies within the crown as seen on bitewing radiograph, i.e. six teeth surfaces (35.3%) showed radiolucency in the outer half of the enamel, three teeth surfaces (50.0%) showed radiolucency in both the outer and inner halves of the enamel. Twenty-three teeth surfaces (17.4%) showed no radiolucency on bitewing radiograph and 2 teeth surfaces (14.3%) showed radiolucency extending beyond the dentinoenamel junction suggestive of dentinal caries. The sensitivity of the DIAGNOdent technique to bitewing radiography in diagnosing enamel caries was 39.12% and the sensitivity was 84.08%.

**Table 6:** **Table 6:** Caries distribution as per radiographic interpretation

*Score*		*Caries level*		*Criteria*		*No. of teeth*	
R0		D0		No radiolucency		132	
R1		D1		Zone of increased radiolucency, confined to outer half of enamel		17	
R2		D2		Zone of increased radiolucency involving both inner and outer halves of enamel layer, including lesions extending up to but not beyond the DEJ		6	
R3		D3		Zone of increased radiolucency penetrating the enamel and DEJ and progressing into the dentin		14	
Total						169	

**Table 7:** **Table 7:** Correlation between DIAGNOdent and bitewing radiographs

						*Bitewing radiograph*									*Total*	
						*R0*		*R1*		*R2*		*R3*				
DIAGNOdent		Sound		Count		101		7		1		1			110	
				%		76.5%		41.2%		16.7%		7.1%			65.1%	
		Enamel caries		Count		23		6		3		2			34	
				%		17.4%		35.3%		50.0%		14.3%			20.1%	
		Dentinal caries		Count		8		4		2		11			25	
				%		6.1%		23.5%		33.3%		78.6%			14.8%	
Total				Count		132		17		6		14			169	
				%		100%		100%		100%		100%			100%	

c^2^ = 66.4; p < 0.001; VHS: very high significant

Of the 25 teeth surfaces (14.8%) scored as dentinal caries (D3) by the DIAGNOdent, 11 teeth surfaces (78.6%) were found to have radiolucencies extending beyond the dentinoenamel junction as seen on bitewing radiographs. Eight teeth surfaces (6.1%) showed no radiolucency in radiograph, four teeth surfaces (23.5%) showed radio-lucency confined to the outer half of the enamel and two teeth surfaces (33.3%) showed radiolucency involving both the inner and the outer halves of the enamel. The sensitivity of the DIAGNOdent technique to bitewing radiography in diagnosing dentinal caries was 78.57% and the specificity was found to be 90.97%.

The Chi-square test showed a strong association between the DIAGNOdent and the bitewing radiography (p < 0.001).

## DISCUSSION

Considering the general decrease in the prevalence of dental caries throughout the world, the employment of diagnostic thresholds that permit the early detection of pathological alteration in dental mineralized tissue has become desirable.^20^ More sensitive diagnostic methods and criteria, including the recording of non-cavitated lesions are necessary.^21,22^ If an initial lesion is detected before the cavitation stage, it can be arrested easily^23^ and probably will prevent future invasive treatment and more serious damage of the dental tissues.^23-26^ Based on such criteria, some authors have defended the investigation of methods for early detection of caries.^20,25^

Concerning the DIAGNOdent, studies have demonstrated that the device is able to detect early caries lesions.^27-29^ An *in vitro* study showed better results regarding the performance of the DIAGNOdent in detecting occlusal caries lesions in advanced enamel caries lesions (D2).^28^ Another *in vitro* study comparing DIAGNOdent and bitewing radiography in detection of proximal caries in primary teeth demonstrated that the reliability of DIAGNOdent was very high and its diagnostic validity was higher than that of bitewing radiography.^10^ A systematic review reported that there is a lack of evidence related to caries detection in primary teeth.^30^

In the present study, comparisons among DIAGNO-dent and bitewing radiography in detection of proximal caries in primary teeth *in vivo* was carried out, and it was observed that the DIAGNOdent presented better performance when used at the D3 threshold (78.6%). As the DIAGNOdent device measures the fuorescence from the organic contents of the carious lesions, the device is expected to perform better at dentin threshold than at the enamel threshold.^20,28^

Following the detection of caries at the D3 threshold, the sensitivity of the DIAGNOdent was better at the D0 threshold (76.52%). This finding is in agreement with other studies^27,31-33^ conducted for the detection of caries on occlusal surface, which reported a substantial sensitivity using the DIAGNOdent. The advantage of detecting demineralization at this stage allows the early intervention for caries reversal.

At the enamel threshold (D1 and D2), the sensitivity was found to be 39.12%. Within the enamel caries (D1 and D 2), detection at the D2 threshold (35.3%) was better than detection of caries confined to the outer half of the enamel (D1) (50%).

The overall result shows that the DIAGNOdent can be used as an alternative diagnostic method in detection of proximal caries in primary teeth with the added advantage that there is no exposure to harmful radiation and allowing continuous monitoring of lesions at regular intervals.

When seen at the each caries level, the DIAGNOdent is more accurate at the D0 and D3 threshold.

## CONCLUSION

The DIAGNOdent can be used as an alternative method for detection of proximal caries in children.

The DIAGNOdent device performs better at the dentin (D3) threshold followed by D 0 threshold. However, the device does not show good performance in detecting enamel caries (D1 and D2), particularly the initial enamel caries lesions in the proximal surface of primary teeth.
